# Molecular Characteristics of the Malate Dehydrogenase (MDH) Gene Family in *Spirometra mansoni* (Cestoda: Diphyllobothriidea)

**DOI:** 10.3390/ijms25168802

**Published:** 2024-08-13

**Authors:** Ruijie Wang, Jie Hao, Chengyue Cao, Jing Li, Xi Zhang

**Affiliations:** Department of Parasitology, School of Basic Medical Sciences, Zhengzhou University, Zhengzhou 450001, China; 17839975524@163.com (R.W.); hj17839969640@163.com (J.H.); ccy20010925@163.com (C.C.); lijinghello@126.com (J.L.)

**Keywords:** tapeworm, *Spirometra mansoni*, malate dehydrogenase, gene expression, molecular characterization

## Abstract

The plerocercoid larva of *Spirometra mansoni* can cause a parasitic zoonosis—sparganosis. Malate dehydrogenase (MDH) plays a very important role in the life activities of parasites. However, little is known about the MDH family in *S. mansoni*. We identified eight new MDH members in *S. mansoni* in this study. Clustering analysis divided *Sm*MDHs into two groups and revealed patterns similar to the conserved motif organization. RT–qPCR suggested that five MDHs were highly expressed in the mature proglottid and that three MDHs were highly expressed in the gravid proglottid. Phylogenetic analysis revealed that *Sm*MDHs contain both conserved family members and members in the process of further diversification. r*Sm*MDH has an NAD binding domain, a dimer interface and a substrate binding domain. Natural *Sm*MDH was immunolocalized in the tissues and follicles around the uterus in the mature or gravid proglottid and eggshells. The maximum forward and reverse reaction activities of r*Sm*MDH were observed at pH 8.5 and 9.0, respectively. The optimum temperature for enzyme activity was 37 °C in the forward reaction and 40 °C in the reverse reaction. These results lay the foundation for studying the molecular functions and mechanisms of MDHs in *S. mansoni* and related taxa.

## 1. Introduction

The motile larvae (plerocercoid) of the *Spirometra mansoni* tapeworm can cause sparganosis. The means of human infection includes eating undercooked infected frogs and snakes and drinking water with infected copepods. After infecting humans, the ingested plerocercoid can invade the subcutaneous tissues, eyes and brain, resulting in local tissue damage, blindness, and even death. More than 2000 human sparganosis cases have been reported thus far, with the majority coming from Asian countries, especially in China, Korea, and Japan [1]. However, with respect to medical damage, knowledge about the molecular mechanism underlying the growth and development of *S. mansoni* is still limited [2]. Therefore, we are committed to reducing this knowledge gap.

Our previous joint analysis of the transcriptome and proteome of plerocercoid and adult *S. mansoni* revealed that malate dehydrogenase (MDH) is a multi-copy and highly expressed family, suggesting potentially significant roles for MDH in the growth and development of *Spirometra* tapeworms [3]. MDH is a polysomic enzyme belonging to the lactate dehydrogenase (LDH)/MDH superfamily. It is ubiquitous in organisms, usually in the form of LDH (tetrameric enzyme), LDH-like MDH (tetrameric enzyme) and MDH (dimeric enzyme) [4,5]. During cellular energy metabolism, MDH reversibly catalyzes the conversion between malate and oxaloacetate by using NAD+ and NADH as cofactors. MDH contains two isoforms, mitochondrial MDH (mMDH) and cytoplasmic MDH (cMDH). mMDH is involved in aerobic oxidation and cMDH transfers reducing equivalents in the form of malate/oxaloacetate [4,6,7,8]. cMDH also participates in gluconeogenesis [9], the glyoxylate cycle [10], glyoxylate degradation [11], and mixed acid fermentation [12]. In *Escherichia coli*, the existence of MDH helps *E. coli* to cope with oxidative stress, suggesting a key role of MDH in bacteria [13,14]. Both cMDH and mMDH are helpful in adjusting the energy metabolism of parasitic worms [15,16]. cMDH participates in anaerobic metabolism in the host bile duct of fluke parasites [17]. cMDH is often highly expressed in organs that require high ATP levels and is involved in the central metabolism between organelle compartments [18,19]. It removes NADH from the oxidation of cytosolic substrates via malate-aspartate shuttling and participates in and maintains redox homeostasis [20].

Owing to its important roles in both anaerobic and aerobic oxidation in helminths, malate dehydrogenase has emerged as a target for vaccine development and drug screening [21]. The presence of mMDH in both infected and noninfected *Trypanosoma cruzi* strains can provide new ideas for the development of new therapies for preventing *T. cruzi* infection [22]. The analysis of the molecular characteristics of malate dehydrogenase in *Taenia solium* helps us better understand the biological characteristics of *T. solium* and lays the foundation for a deeper knowledge of the role of malate dehydrogenase in the pathogenesis of *T. solium* [23]. However, the research on the *S. mansoni* malate dehydrogenase family’s polymorphisms and the molecular signature is limited. In this study, we first explored the variety of MDH family members in *S. mansoni* and the phylogenetic patterns of MDHs in platyhelminths, after which the molecular signature of malate dehydrogenase was further investigated.

## 2. Results

### 2.1. MDHs in Medical Platyhelminths

**Figure 1:**
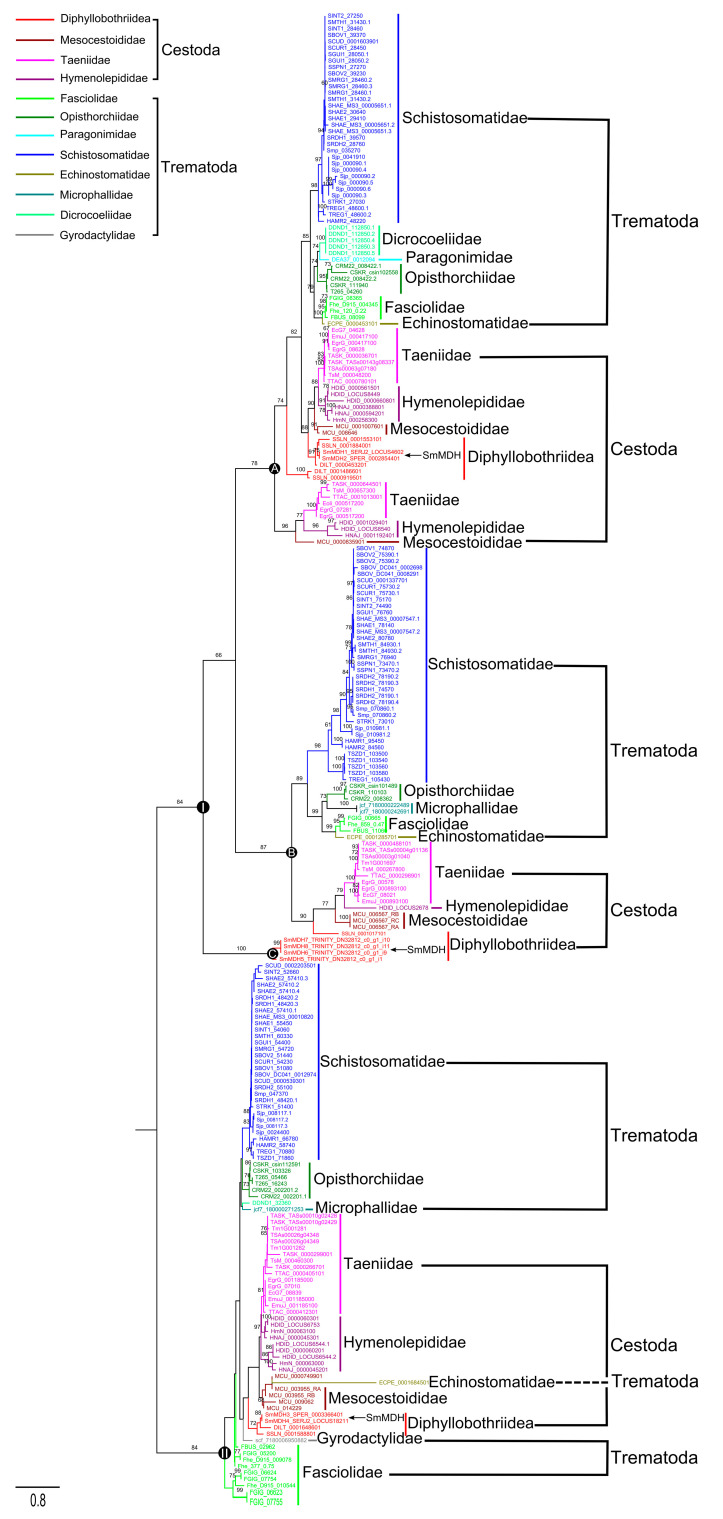
Phylogenetic analysis of malate dehydrogenase in medical cestodes and trematodes based on the maximum likelihood method. The values on the branches represent bootstrap values, and only values with bootstrap values greater than 60 are presented.

A total of 232 MDH sequences from 16 cestodes and 26 trematodes were retrieved (Supplementary Table S1). The tree topologies of cestode and trematode obtained were consistent using both ML and BI methods. Here, the phylogenetic relationships of medical platyhelminth MDHs were presented using only the ML tree. The cestodes and trematodes were mixed in clade I and clade II (Figure 1). Specifically, three main groups—A, B and C—appeared in clade I. In group A, trematodes from six families clustered together—*Schistosomatidae*, *Dicrocoeliidae*, *Paragonimidae*, *Opisthorchiidae*, *Fasciolidae*, and *Echinostomatidae*—while some cestodes (including *Taeniidae*, *Hymenolepididae*, *Mesocestoididae* and *Diphyllobothriidea*) were relatively dispersed. Within group B, the fluke and tapeworm groups formed two stable branches. However, group C contained only four members from *Diphyllobothriidea*. Compared with those in clade I, the distributions of species in clade II were more chaotic: some flukes from *Schistosomatidae*, *Opisthorchiidae*, *Dicrocoeliidae* and *Microphallidae* formed a relatively independent group, while the remaining tapeworms and flukes were scattered. Interestingly, although the MDHs of medical platyhelminths showed high sequence diversity, the MDHs from the same family tended to cluster together, indicating a certain degree of conservation of MDHs. For the MDH sequence in *S. mansoni*, *Sm*MDH1 and *Sm*MDH2 were distributed in group A of clade I, *Sm*MDH5-8 formed group C, and *Sm*MDH3 and *Sm*MDH4 were inserted into clade II.

### 2.2. Manual Annotation of SmMDH Genes

**Figure 2:**
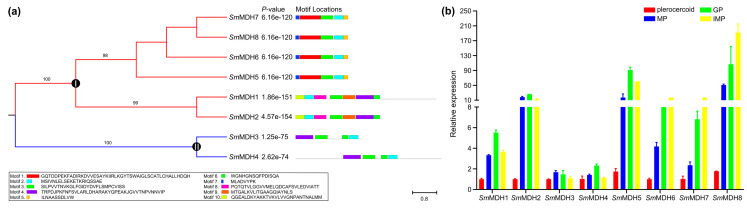
Gene clustering and expression pattern analysis of *S. mansoni*. (**a**) Cluster analysis and conserved motifs of eight *Sm*MDHs. According to the phylogenetic results, it can be divided into clade I and clade II. The numbers on the branches are bootstrap values, and only values above 60 are shown. (**b**) MDH gene expression of *S. mansoni* in different stages determined using qRT–PCR. Red represents the plerocercoid, blue represents the MP, green represents the GP, and orange represents the IMP. IMP: immature proglottid; MP: mature proglottid; GP: gravid proglottid. GAPDH was used as an internal reference gene. The expression level was measured with the 2-ΔΔCt method. The data were averaged from three repeats, The error bars represent the SDs (n = 3).

Eight MDH members were determined in *S. mansoni*. *Sm*MDH and *Sm*MDH3 were identified in the WormBase ParaSite database, four sequences (*Sm*MDH5-*Sm*MDH8) were identified from our RNA sequencing data (PRJNA761840), and the remaining two sequences (*Sm*MDH1 and *Sm*MDH4) were identified from the proteomic data (PXD039620). The length of these *Sm*MDHs ranged from 896 bp to 12708 bp. The predicted amino acid ranged from 123 aa to 343 aa. The domain lengths ranged from 42 aa to 263 aa (Table 1). Clustering analysis divided the *Sm*MDHs into two groups: group I and group II. Six *Sm*MDHs belong to group I, while group II contained two members (Figure 2a). The MEME identified 10 specific motifs that contained 8 to 50 residues (Table S2). To determine the expression patterns of the identified *Sm*MDHs, we sampled plerocercoids and adult worms for analysis via qRT–PCR (Figure 2b and Table S3). In both the plerocercoid and adult stages, eight *Sm*MDHs were expressed. In the adult stage, eight genes were highly expressed. Among these genes, the highly expressed gene numbers in the mature proglottid and gravid proglottid are 5 and 3, respectively.

### 2.3. Cloning, Expression and Identification of rSmMDH1

Due to its high expression level and strong interaction with other proteins, *Sm*MDH1 was selected for molecular characterization. Molecular biological analysis revealed that MDH is a cytoplasmic protein, with an Mw of 35.76935 ku and an isoelectric point of 8.20 that contains a dimer interface, NAD+ binding sites and a malate binding site. The 3D structure showed the typical N-terminal and C-terminal domains in MDH. Pymol was used to visualize the MDH binding site and the results reveal that 11 (GLY), 13 (ALA), 14 (GLY), 15 (GLN), 16 (ILE), 42 (ASP), 87 (VAL), 88 (GLY), 89 (ALA), 90 (MET), 108 (ILE), 129 (VAL), 131 (ASN), 155 (LEU), 187 (HIS) and 241 (SER) were the NAD binding sites; 18 (TYR), 55 (MET), 56 (GLU), 58 (GLN), 59 (ASP), 60 (CYS), 161 (ASN), 162 (ARG), 165 (ALA), 230 (ARG), 233 (ALA), 237 (ALA), 238 (ARG), 242 (SER), 243 (ALA), 244 (ALA), 245 (SER) and 248 (LYS) were the dimer interfaces; and 92 (ARG), 98 (ARG), 131 (ASN), 158 (LEU), 162 (ARG), 187 (HIS), 235 (ILE) and 242 (SER) were the malate binding sites (Supplementary Figure S1).

**Figure 3:**
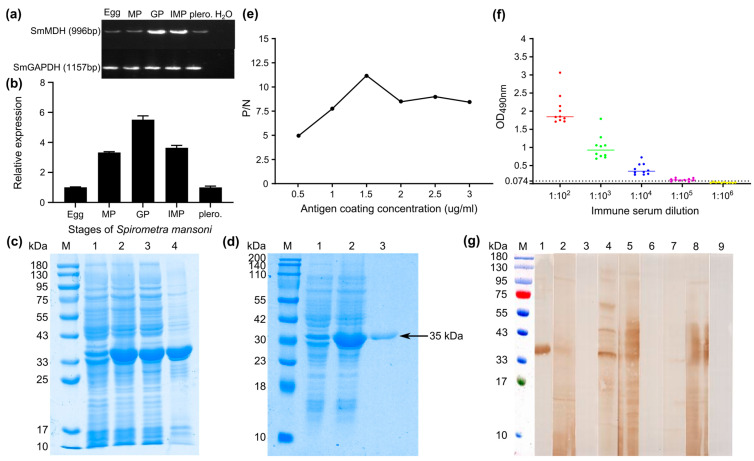
Molecular characterization of the cloned *Sm*MDH. (**a**,**b**) The transcription pattern of the MDH gene in various developmental stages of *S. mansoni* including eggs, plerocercoids and adults. Conventional RT–PCR (**a**) and real-time RT–PCR (**b**) were performed. A housekeeping gene (GAPDH) was used as an internal reference. H_2_O was used as a negative control. IMP: immature proglottid; GP: gravid proglottid; MP: mature proglottid; Plero: plerocercoid. (**c**) soluble r*Sm*MDH1 analysis. M: protein prestaining marker; Lane 1: uninduced bacterial cultures; Lane 2: the lysate of the induced recombinant bacteria harboring pQE-80L-r*Sm*MDH1 after ultrasonication; Lane 3: the supernatant protein; Lane 4: the precipitate protein; (**d**) SDS–PAGE analysis after r*Sm*MDH1 purification. M: protein prestaining marker; Lane 1: uninduced bacterial cultures; Lane 2: the lysate of the induced recombinant bacteria harboring pQE-80 L-r*Sm*MDH1 after ultrasonication; Lane 3: r*Sm*MDH1 purified by a Ni–NTA–sefinose column. (**e**) Determination of the optimal antigen coating concentration. (**f**) Anti-r*Sm*MDH immunoserum potency assay. Red, blue, green, purple, yellow, and cyan represent serum dilutions of 1:10^2^, 1:10^3^, 1:10^4^, 1:10^5^, 1:10^6^, and 1:10^7^, respectively. (**g**) r*Sm*MDH1 antigenicity analysis. M: Protein prestained marker; 1: r*Sm*MDH1 + anti-r*Sm*MDH1 serum; 2: r*Sm*MDH1 + infected mouse serum; 3: r*Sm*MDH1 + normal mouse serum; 4: soluble antigen + anti-r*Sm*MDH1 serum; 5: soluble antigen + serum of infected mice; 6: soluble antigen + normal mouse serum; 7: ES antigen + anti-r*Sm*MDH1 serum; 8: ES antigen + infected mouse serum; and 9: ES antigen + the serum of normal mice.

**Figure 4:**
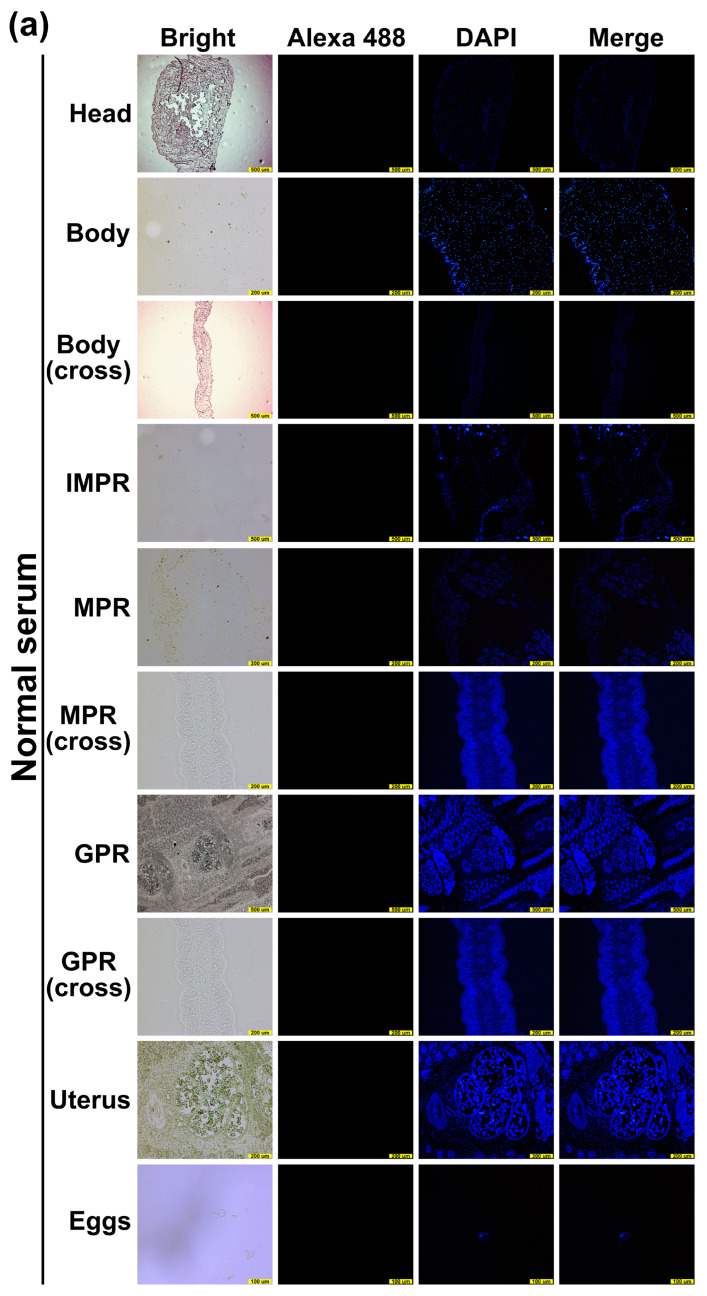

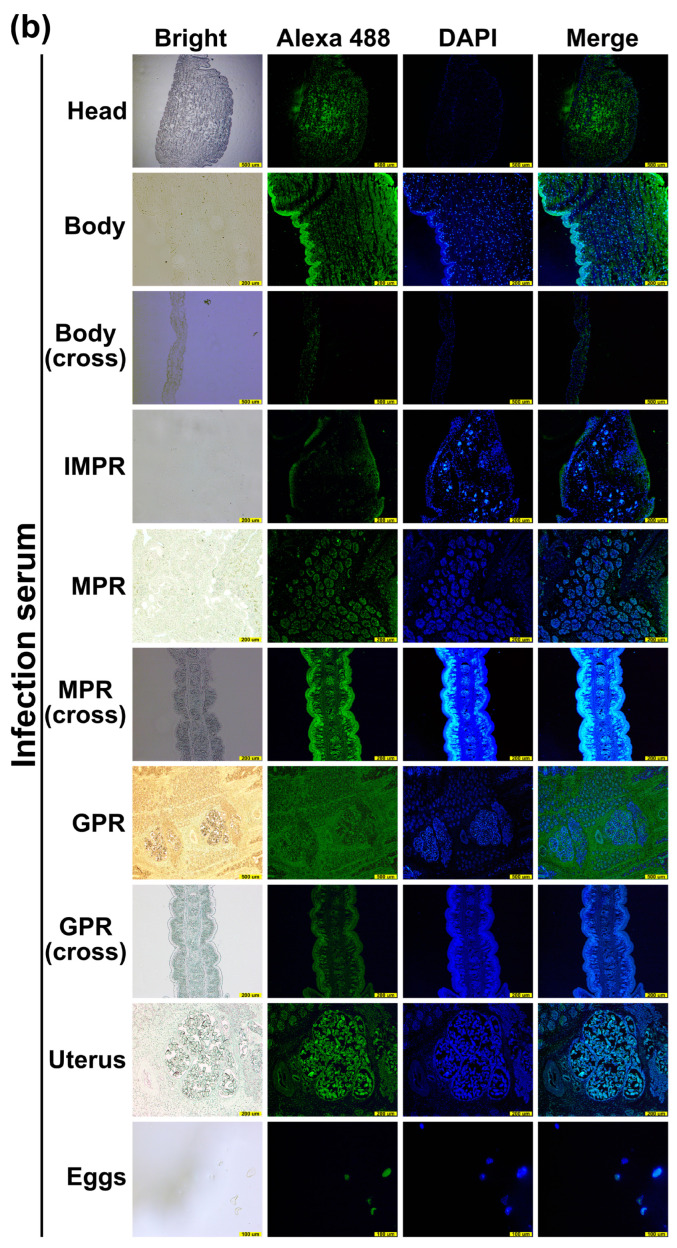

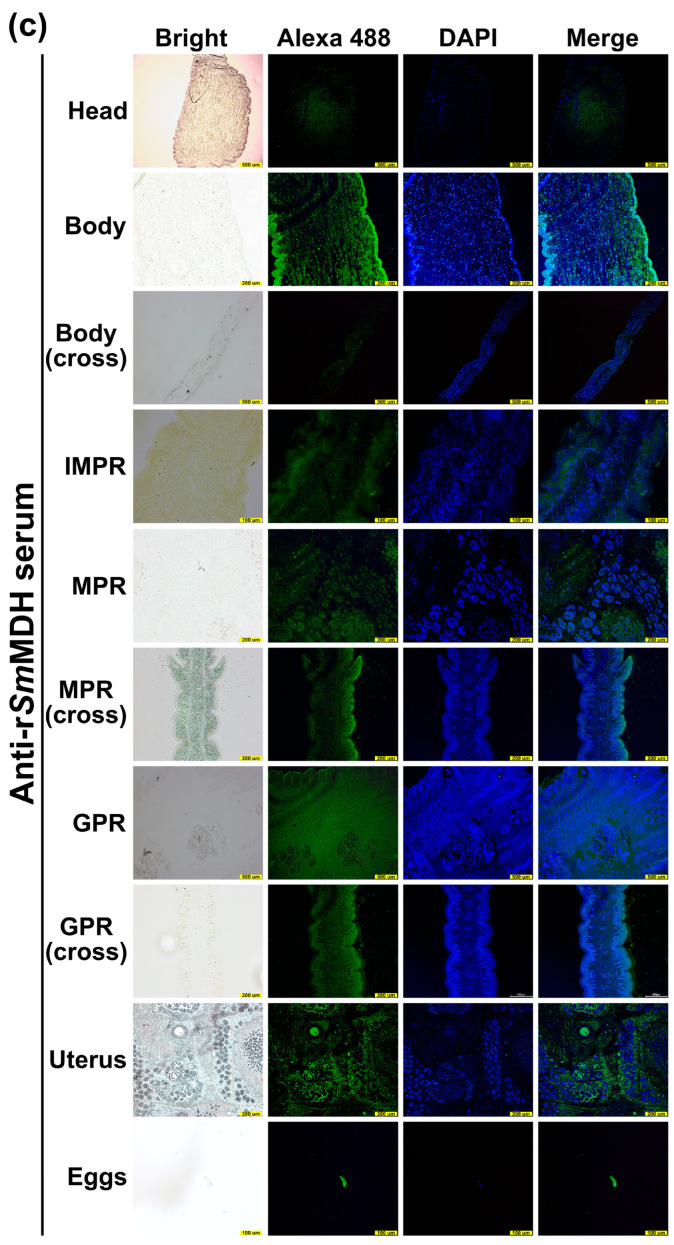
Immunofluorescence analysis of MDH in various stages of *Spirometra mansoni*. Head: head of plerocercoid; body: body of plerocercoid; body (cross): body of plerocercoid cross; MPR: mature proglottid; MPR (cross): mature proglottid cross; GPR: gravid proglottid; GPR (cross): gravid proglottid cross; IPR: immature proglottid; Egg: eggs in the uterus of gravid proglottid. (**a**) Normal serum; (**b**) infected serum; (**c**) anti-r*Sm*MDH serum. GPR, scale of body (cross), head: 500 µm; IMPR, MPR, MPR (cross), GPR (cross), Uter, body: 200 µm; Eggs: 100 µm.

mRNA transcription (996 bp) of the *Sm*MDH1 gene was detected at the egg, plerocercoid and different proglottid stages in adult worms (Figure 3a). qPCR analysis revealed that the transcriptional level in the gravid proglottid stage was the highest, followed by that in the immature proglottid and the plerocercoid stages (Figure 3b). r*Sm*MDH1 was purified using Ni 2+ affinity chromatography and identified with SDS–PAGE (Figure 3c,d). The optimal protein concentration was 1.5 µg/mL and 1:100 was the best mouse serum dilution (Figure 3e). A cut-off value of standard for subsequent tests was 0.074 (Figure 3f). Western blotting analysis revealed that r*Sm*MDH1 was recognized by the anti-r*Sm*MDH1 serum, soluble antigen and ES antigen (Figure 3g). The immunolocalization test revealed specific fluorescence staining in eggshells, plerocercoids and adults. r*Sm*MDH1 was concentrated in the epidermis and parenchyma of plerocercoid and adult, and significant fluorescence was observed in the tissues and follicles around the uterus in the mature or gravid proglottid and eggshells, which indicates that malate dehydrogenase was highly expressed in the uterus and eggshell (Figure 4).

### 2.4. Enzyme Kinetics

**Figure 5:**
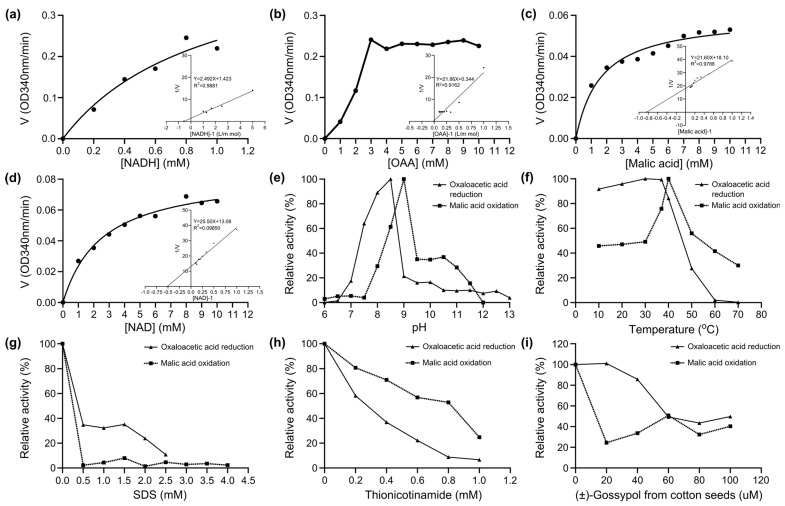
Enzymatic characteristics of *Sm*MDH. (**a**) Kinetic study of the effects of the substrate concentration of NADH on the enzymatic activity of r*Sm*MDH1. The kinetic parameters Km and Vmax were determined using Lineweaver–Burk plots. The Km and Vmax were 1.7507 mM and 0.9269 μmol min^−1^ mL^−1^, respectively. (**b**) Kinetic study of the effects of OAA substrate concentration on r*Sm*MDH1 enzymatic activity. The Km and Vmax were 63.53 mM and 3.73 μmol min^−1^ mL^−1^, respectively. (**c**) Kinetic study of the effects of the malic acid concentration on the enzymatic activity of r*Sm*MDH1. The Km and Vmax were 1.1933 mM and 0.071 μmol min^−1^ mL^−1^, respectively. (**d**) Kinetic study of the effects of the substrate concentration of NAD on the enzymatic activity of r*Sm*MDH1. The Km and Vmax were 1.9489 mM and 0.098 μmol min^−1^ mL^−1^, respectively. (**e**) Effects of pH and temperature on r*Sm*MDH1 enzymatic activity. (**f**) Effects of temperature on r*Sm*MDH1 enzymatic activity. (**g**) Kinetic study effects of sodium dodecyl sulphate (SDS). (**h**) Kinetic study effects of thionicotinamide. (**i**) Kinetic study effects of (±) gossypol from cotton seeds.

**Figure 6:**
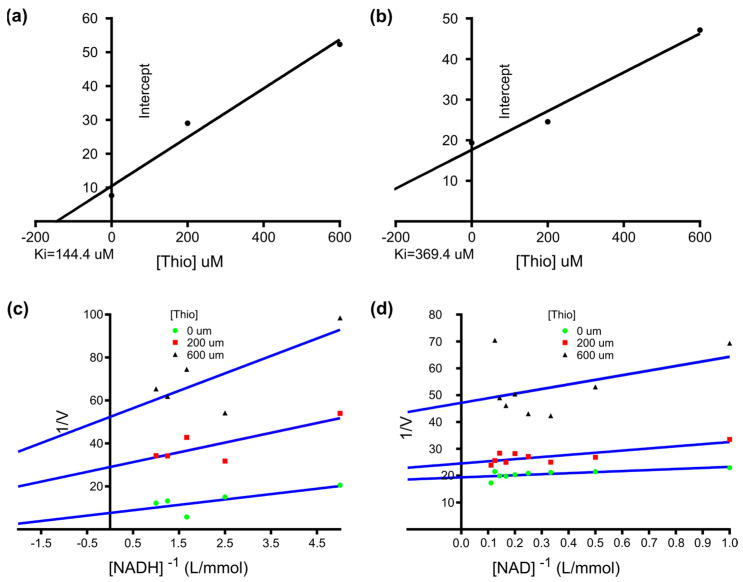
Effects of different concentrations of thionicotinamide (Thio) on r*Sm*MDH1 enzyme activity. Figure (**a**,**c**) show the oxalacetic acid oxidation of r*Sm*MDH1. (**a**) The effect of different concentrations of thionicotinamide (Thio) on the initial velocities. (**c**) Thio (200, 600 µM). Figure (**b**,**d**) show the malic acid reduction of r*Sm*MDH1. (**b**) The effect of different concentrations of thionicotinamide (Thio) on the initial velocities. (**d**) Thio (200, 600 µM). The inset shows a secondary plot of the 1/Vmax values derived from the primary Lineweaver–Burk plot vs. concentration for the determination of Ki.

A kinetic study with r*Sm*MDH1 showed that increasing the NADH concentration (0.2–1.0 mM) slightly increased the activity of MDH (Figure 5a). Then, it increased to a certain level and reached the saturation point at 0.8 mM of NADH. The saturation points of OAA (1–10 mM) (Figure 5b), malic acid (1–10 mM) (Figure 5c) and NAD (1–10 mM) (Figure 5d) were at 9 mM, 10 mM, and 8 mM, respectively. The maximum forward and reverse reaction activities were observed at pH 8.5 and 9.0, respectively (Figure 5e). The optimum temperature for enzyme activity was 37 °C in the forward reaction and 40 °C in the reverse reaction (Figure 5f). SDS strongly inhibited the enzyme: when the concentration reached 2.5 mM the forward and reverse reactions were almost completely inhibited because SDS was able to destroy the hydrogen and hydrophobic bonds in the enzyme protein molecules, change the spatial conformation of the enzyme, and reduce the enzyme activity (Figure 5g). The effect of thionicotinamide (Thio) on the positive reverse reaction enzyme activity showed a clear dose-dependent effect (Figure 5h). With increasing compound concentration gossypol inhibited both the forward and reverse reactions (Figure 5i). Plotting the 1/v (µM min^−1^) axis intercept against the inhibitor concentration yielded *Ki* values of 144.4 µM and 369.4 µM for Thio in the forward and reverse reactions, respectively (Figure 6).

**Figure 7:**
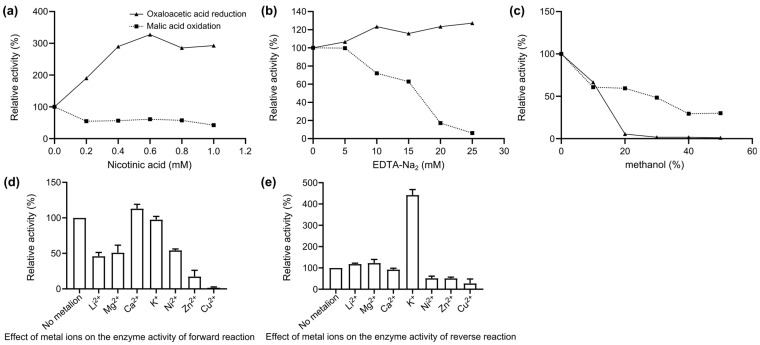
Kinetic study of the effects of different compounds and metal ions. (**a**) Kinetics of nicotinic acid. (**b**) Kinetic study of the effects of an ethylenediaminetetraacetic acid disodium salt solution (EDTA-Na2). (**c**) Kinetic study of methanol. (**d**) Effect of different metal ions on the enzyme activity of the forward reaction. (**e**) Effect of different metal ions on the enzyme activity of the reverse reaction.

Nicotinic acid clearly promoted the forward reaction and inhibited the reverse reaction (Figure 7a). EDTA-Na_2_ activated the forward reaction of MDH to some extent, with obvious inhibition of the reverse reaction (Figure 7b). Methanol inhibited both the forward and reverse reactions but more strongly inhibited the forward reaction (Figure 7c). Li^2+^ and Mg^2+^ inhibited the positive reaction but promoted the reverse reaction, Ca^2+^ had little effect on the enzyme activity, K^+^ significantly activated the reverse reaction, and Ni^2+^, Zn^2+^, and Cu^2+^ strongly inhibited both the forward and reverse reactions (Figure 7d,e).

## 3. Discussion

Malate dehydrogenase catalyzes the reversible reaction between malate and oxaloacetate in which mitochondrial malate dehydrogenase mainly catalyzes the oxidation of malate and participates in the tricarboxylic acid cycle, while cytoplasmic malic acid dehydrogenase mainly catalyzes the reduction of oxaloacetate. The main role of the malate/aspartate shuttle involved in cytoplasmic malate dehydrogenase is to transport reductive equivalents and metabolites at the mitochondrial membrane and to regulate the citric acid cycle [24]. Thus, cytoplasmic malate dehydrogenase is a key checkpoint for the energy balance between mitochondria and the cytoplasm [25,26]. MDH plays a crucial role in seed germination [27], cell growth [28], embryonic development [29], plant development and maturation [30], and stress resistance [31,32]. With few studies having been undertaken on parasite malate dehydrogenase, it is unclear whether it plays an important role in parasite growth and development. Therefore, in this study, the identification, evolution pattern, clonal expression and molecular characteristics of malate dehydrogenase (*Sm*MDH1) provided a foundation for an exploration of the molecular biological mechanism of MDH in *S. mansoni* and its role in its growth and development.

Searches of the WormBase ParaSite database and omics data identified eight members of the *Sm*MDH gene family, and analysis of the intron/exon distribution of the eight *Sm*MDH family members revealed that they are evolutionarily relatively conserved and likely of the same origin. Quantitative analysis of *Sm*MDH expression at different stages revealed that all eight genes were highly expressed in the adult, suggesting that MDH genes play a large role in the adult stage and may be involved in the growth and development of *S. mansoni*. The 3D structure and the active site of MDH are more conserved. The NAD+-binding site consists of two β folds, three α-helices, and five irregular coils. The substrate binding site includes three irregular coils and four α-helices. The dimer interface consists of an interacting α-helix. The spatial structure of proteins can affect their function and the α-helix can stabilize the skeleton. The enzyme active site often consists of β-folding and irregular coils. Irregular coils can also stabilize the internal structure of the protein, increasing its tolerance to some stimuli [33,34]. Because the MDH structure mainly contains α-helices and irregular coils it is speculated that MDH may have increased tolerance. The active site of MDH includes a hydrophobic cavity with a substrate and a coenzyme-specific binding site. This cavity underwent a conformational change during the formation of the enzyme–coenzyme–substrate ternary complex to prevent the active site from contacting the solvent, reduce the distance between the active site and the substrate, help convert the substrate into a product, and allow the enzymatic reaction to proceed smoothly [7,35].

Due to the clear genetic background of the *E. coli* system, its wide application, low cost, and high availability of target proteins in a short time, it was used in this study for the clonal expression of recombinant proteins. However, the prokaryotic expression system is unstable and susceptible to induction conditions [36], so we optimized the temperature, time and IPTG concentration to obtain the maximum amount of protein. We found that the maximum protein expression could be obtained after 4 h at 37 °C and an IPTG concentration of 0.5 mM, so the above conditions were determined to be the optimal induction conditions. Two forms of protein—soluble fusion protein and insoluble inclusion body protein—were obtained from the *E. coli* expression system. After induction, the recombinant MDH protein was obtained from the supernatant and precipitated. Because the activity of the soluble fusion protein was basically unaffected and the purification operation was simple, the supernatant soluble fusion protein was selected for subsequent study. The purified protein electrophoresis results that were obtained indicate a successful purification of the target protein with the corresponding molecular weight. To test the antigenicity of the protein and its recognition ability, ELISA and Western blotting were performed and the results reveal that the recombinant protein was immunogenic, stimulating mice to produce an immune response to obtain a higher serum titre. Serum obtained from immunized mice specifically recognized recombinant protein and soluble antigens but poorly recognized ES antigens, suggesting that this protein is expressed in the plerocercoid tract but is not highly expressed in its excreta.

The N-terminal sequence, specific protein motifs, amino acid composition, and regulation of complex life activities may affect the localization of proteins [37]. Thus, the localization of MDH varies in different life history periods. The IFA results show that MDH is mainly present in the cortex of the plerocercoid, the eggshell, and the uterine and parenchymal tissues. This suggests that the regulation of energy balance by *S. mansoni* changes with the life history period. The conserved C-terminal domain in different MDH proteins acts as a redox switch to regulate enzymatic activity [38]. Previous studies proposed four competing mechanisms for pH-dependent effects based on kinetic data from MDH, including the partial random ordered bibi mechanism [39], the formation of an abortive complex [40] at higher pH, the reciprocating forced order mechanism [41], and the ordered bibi mechanism [42,43], which form several abortive complexes. It is widely accepted that the kinetics of the two malate dehydrogenase isoforms follow an ordered mechanism in which MDH is first bound to a coenzyme (NAD/NADH) and then bound to a substrate [44,45,46]. Chapman [47] reported that OAA is not only a specific substrate for MDH but also that the MDH conformation changes only after proton transfer from NADH to OAA. This also suggests that the reaction is an ordered reaction that follows an ordered mechanism. Because the reaction catalyzed by malate dehydrogenase involves the reversible exchange of protons, the pH and ionic strength of the solvent influence the catalytic activity of MDH. High temperature will destroy the structure of the protein, causing the protein to lose its original function. As the temperature increases, the catalytic activity of MDH continuously decreases until it completely loses its activity. In this study, the results show that the recombinant protein MDH catalyzed the positive reaction at a greater rate than did the reverse reaction. The localization analysis showed that MDH is mainly present in the cytoplasm, which is consistent with the finding from other studies [25]. Different inhibitors have different types of MDH inhibition, in which thionicotinamide has an obvious dose dependence on MDH, so *Km* and *Vm* were calculated and both were changed, indicating that thionicotinamide inhibited the catalytic reaction of MDH by binding to the enzyme-substrate complex, a reversible inhibitor.

## 4. Materials and Methods

### 4.1. Ethical Approval

The study was conducted in accordance with the guidelines of the Declaration of Helsinki, and all procedures involving animals were approved by the Life Science Ethics Committee of Zhengzhou University (permit code SYXK 2021-7023). The animals were handled in accordance with good animal practices, as required by the Animal Ethics Procedures and Guidelines of the People’s Republic of China.

### 4.2. Samples and Experimental Animals

The plerocercoids were collected from infected frogs (*Pelophylax nigromaculatus*) from Zhengzhou city in central China [48] and identified as *Spirometra mansoni* by molecular typing using the method described in [49]. As described previously, an adult cestode was obtained from an infected domestic cat [50]. The immature proglottid (IP), mature proglottid (MP) and gravid proglottid (GP) of adult were collected according to their position and morphology [51]. Twenty female kunming mice (4~6 weeks old) were immunized by antigens of recombinant malate dehydrogenase (rMDH) four times to obtain the anti-r*Sm*MDH serum. All anti-r*Sm*MDH serum were stored at −80 °C until use. All samples were washed thoroughly with physiological saline, snap-frozen in liquid nitrogen and stored at −80 °C for further use.

### 4.3. SmMDH Family Member Identification

Candidate sequences that contain the malate dehydrogenase protein domain were searched using the NCBI Conserved Domains database (www.ncbi.nlm.nih.gov/Structure/cdd/wrpsb.cgi accessed on 4 January 2024). All candidate *Sm*MDHs were obtained from the WormBase ParaSite database (https://parasite.wormbase.org/ accessed on 4 January 2024) and recently published transcriptomic data and proteomic data [2,52]. These extracted sequences were identified as members of the MDH family through querying for genes annotated with the Pfam domain accession pfam0056, pfam02866, pfam03435 and pfam01073. All screened candidates from multiomic data were analyzed using the HMMER tool to confirm the presence of the conserved malate dehydrogenase [53]. In addition, for candidate *Sm*MDHs retrieved from the transcriptomic data, the nucleotide sequences were firstly translated to amino acid sequences using the NCBI’s ORF finder tool (https://www.ncbi.nlm.nih.gov/orffinder/ accessed on 5 January 2024) and using BLASTX for homology searches. Finally, these retrieved sequences were corroborated by the cloning and sequencing of *S. mansoni* DNAs. The basic physical and chemical properties of all identified *Sm*MDHs were predicted using the ExPASy (https://www.expasy.org/ accessed on 5 January 2024) and NCBI servers. The subcellular localization was predicted by TargetP (www.cbs.dtu.dk/services/TargetP/ accessed on 6 January 2024). The conserved protein motif analysis was performed using the mixture model by expectation maximization (MEME) method, and the gene features were performed using GSDS. Motif scan and NCBI-CDD server were used for conserved functional protein domain prediction. Multiple sequence alignments of *Sm*MDHs were carried out with DNAMAN v9.0. Secondary structure prediction was generated by PSIPRED server (http://bioinf.cs.ucl.ac.uk/psipred/ accessed on 6 January 2024). The 3D structure was determined using homology modeling available at the Swiss Model server (https://swissmodel.expasy.org/ accessed on 7 January 2024). The quality of the model was examined using Ramachandran plot analysis in PROCHECK and visualized by the Swiss-Pdb Viewer v.4.1 [54]. Pymol v.1.7 was used to visualize the MDH binding site. The phylogenetic tree was inferred using maximum likelihood (ML) method based on the LG + G model. The ML analysis was performed In MEGA v7 with 1000 bootstrap replications [55].

### 4.4. Quantitative RT-PCR Analysis

Quantitative RT-PCR (qRT–PCR) analysis was performed to monitor the expression levels of identified *Sm*MDHs in two life cycle stages of *S. mansoni*: the plerocercoid stage and the adult stage (including immature proglottid, mature proglottid and gravid proglottid). The gene-specific primers are listed in Table S3. Total RNA was isolated using a reverse transcription kit (Novoprotein, Shanghai, China). qRT–PCR was conducted on a 7500 Fast Real-time PCR system (Applied Biosystem, Monza, Italy). The reaction mixture contained 10 µL of 2 × TB Green Premix Ex Taq (Takara, Beijing, China), 10 µM each of sense and antisense primers, 100 ng of first-strand cDNA. Initial thermal cycling was undertaken at 95 °C for 30 s followed by 40 cycles of 95 °C for 3 s and 60 °C for 30 s. The GAPDH gene served as the internal control [56]. Relative gene expression levels were analyzed according to the comparative 2^-∆∆CT^ method [57].

### 4.5. Phylogenetic Analysis

All available MDH sequences of medical cestodes and trematodes were also extracted from the WormBase ParaSite database. The mixture model by expectation maximization (MEME) method was used to analyze potential motifs [58]. Using MAFFT v7, multiple protein sequence alignment was carried out [59]. Bayesian inference (BI) and maximum likelihood (ML) were used to conduct phylogenetic analysis. BI analysis was performed in MrBayes v.3.2 [60]. The analysis consisted of two runs, each with four MCMC chains running for 5 000 000 generations, and sampling every 100th generation. ML analysis was performed in MEGA v.7.0 [55]. Confidence in each node was assessed by bootstrapping (1000 pseudo-replicates).

### 4.6. Cloning, Expression and Identification of rSmMDH

The *Sm*MDH1 gene deposited in NCBI (GeneBank NO: VZI05048.1, https://www.ncbi.nlm.nih.gov/protein/VZI05048.1/ accessed on 5 January 2024) was amplified by PCR with specific primers carrying BamHI and PstI restriction enzyme sites (Blue font) (forward, 5′-TAGGATCCATGACTGGGGCTTTGAAAGTTCTC-3′, and reverse, 5′-TACTGCAGTCACTTGCAGACCTGAATCG-3′), and the cycling protocol was as follows: 30 cycles of 95 °C for 50 s, 60 °C for 50 s and 72 °C for 50 s. The final PCR products were purified, digested, and cloned into the pQE-80L vector (Ipswich, USA). The recombinant plasmid was then transformed into *Escherichia coli* BL21 (Novagen, La Jolla, CA, USA). Expression of r*Sm*MDH1 was induced by adding 0.5 mM IPTG at 37 °C for 4 h. The r*Sm*MDH1 was purified by Ni 2+ affinity chromatography (Shenggong Biotech, Shanghai, China) and identified by SDS-PAGE. Images of gels were recorded using ImageScanner (GE Healthcare, Fairfield, CT). Another gel was prepared by the same method and used for the Western blotting analysis. Antibody titrations of immunized mice were performed by indirect ELISA. The r*Sm*MDH1 protein was coated onto each well of 96-well plates (BIOFIL, Guangzhou, China) overnight at 4 °C. Protein-coated plates were blocked with 5% skim milk at 37 °C for 2 h. All primary antibodies were diluted with PBS-0.05% Tween 20 (PBST) and added onto precoated ELISA plates with incubation at 37 °C for 2 h. Goat anti-mouse IgG (HRP-labeled)was added at 1: 5000 dilution and incubated for 1 h at 37 °C. Finally, 100 µL OPD chromogen substrate containing H_2_O_2_ were added to each well, the plates were incubated, protected from the light, for 20 min and the reaction was stopped by the addition of 50 µL 2 M H_2_SO_4_. The optical density (OD) of all the wells was measured at 490 nm using a computer-controlled BioTek (Synergy LX, WINOOSKI Contry, VT, USA) microplate reader. Indirect immunofluorescence assay (IFA) was used to locate the positions of the target gene. Eggs, tissue sections of plerocercoids and different proglottids of adult worm were first retrieved after microwaving for 20 min with a 0.01 M citric acid buffer (pH 6.0), blocking with 5% normal goat serum in PBS, then incubated with a 1: 10 dilution of anti-r*Sm*MDH1 serum, serum of mice infected with plerocercoids, serum of cat infected with adults, and normal mouse and cat serum in PBS at 37 °C for 1 h, respectively. The sections were incubated with a 1:100 dilution of FITC-labeled anti-mouse IgG (Santa Cruz, CA, USA), and the nuclei were stained with propidium iodide (PI) at 37 °C for 15 min. Finally, the sections were examined under a fluorescent microscope (Olympus, Tokyo, Japan) after washing 6 times with PBS.

### 4.7. Enzyme Activity Assays

In the forward reaction, 0.8 mM NADH and 1 mM oxaloacetic acid were used as substrates to determine enzyme activity in 100 mM Tris–HCL (pH 7.5), and incubated at 37 °C for 10 min. In the reverse reaction, 5 mM NAD and 10 mM lactate were used as substrates to determine enzyme activity in 100 mM Tris–HCL (pH 8.6), and incubated at 37 °C for 10 min. The effect of temperatures (10, 20, 30, 40, 50, 60 and 70 °C) and pH from 6 to 13 on r*Sm*MDH was determined by keeping the enzyme in different conditions for 10 min, and then enzyme activity was assayed immediately. The absorbance changes were observed at 340 nm. The apparent Km and Vmax values in the kinetic mechanism study were graphically determined using the double-reciprocal Lineweaver–Burk plot. The typical reaction system was supplemented with the chemical compound (sodium dodecylsulfate (SDS), gossypol, thionicotinamide, nicotinic acid, EDTA-Na2, methanol) that would be put to the test. In addition, the inhibitory effect of different metal ions on the r*Sm*MDH enzyme was determined. r*Sm*MDH (25 μg) was incubated with Li^2+^, Mg^2+^, Ca^2+^, K^+^, Ni^2+^, Zn^2+^, and Cu^2+^ for 30 min at 37 °C with Li^2+^, Mg^2+^, Ca^2+^, K^+^, Ni^2+^ and Zn^2+^ concentrations of 10 mM and a Cu^2+^ concentration of 1 mM.

## 5. Conclusions

In this study, eight members of the MDH family were successfully selected, and representative members were identified by clonal expression. The transcript levels and immunofluorescence localization of these genes during different life cycle periods were detected and the results show that r*Sm*MDH may play an important role in the growth and development of *S. mansoni*. In addition, the enzyme kinetic analysis showed that the catalytic efficiency of r*Sm*MDH for the positive reaction was greater than that for the reverse reaction. Different compounds and metal ions inhibited the reduction reaction, and the oxidation reactions were quite different, suggesting that *Sm*MDH may mainly catalyze the positive reaction of oxaloacetate reduction to malate in vivo.

## Figures and Tables

**Table 1:** Annotation features for *Spirometra mansoni* malate dehydrogenase.

Gene Name	Gene ID	MDH Domain Coordinates	Protein Length (aa)	Domain Length (aa)
*Sm*MDH1	SERJ2_LOCUS4602	157–324	331	168
*Sm*MDH2	SPER_0002854401	6–153, 157–198	198	148, 42
*Sm*MDH3	SPER_0003366401	1–61, 63–157	157	61, 95
*Sm*MDH4	SERJ2_LOCUS18211	29–172, 32–116, 174–337	343	144, 85, 263
*Sm*MDH5	TRINITY_DN32812_c0_g1_i1	11–114	123	104
*Sm*MDH6	TRINITY_DN32812_c0_g1_i9	11–114	123	104
*Sm*MDH7	TRINITY_DN32812_c0_g1_i10	11–114	123	104
*Sm*MDH8	TRINITY_DN32812_c0_g1_i11	11–114	123	104

Note: aa indicates the amino acids.

## Data Availability

The data supporting the conclusions of this article are included within the article.

## Supplementary Materials

The supporting information can be downloaded at: https://www.mdpi.com/article/10.3390/ijms25168802/s1.
