# Supplementary material for: Molecular Characteristics of the Malate Dehydrogenase (MDH) Gene Family in Spirometra mansoni (Cestoda: Diphyllobothriidea)
Source: Int J Mol Sci. 2024 Aug 13;25(16):8802. doi: 10.3390/ijms25168802 (PMC11354392; doi:10.3390/ijms25168802)
Supplement: Supplementary file 1 [file ijms-25-08802-s001.zip › ijms-3097202-supplementary.pdf]

Supplementary materials

Molecular characteristics of the malate dehydrogenase (MDH) gene family in *Spirometra mansoni* (Cestoda: Diphyllobothriidea)

Table S1 Summary of malate dehydrogenase for medical tapeworm and trematode.

Table S2. The putative motifs of *Sm*MDH.

Table S3. Primers sequences designed for qRT-PCR.

Figure S1. *Sm*MDH1 structural and functional prediction results. (a) hydrophobicity prediction; (b) transmembrane prediction; (c) functional structure domain prediction; (d) phosphorylation site prediction; (e) signal peptide prediction; (f) secondary structure prediction, the yellow indicates strand, the purple represents helix; (g) 3D structure prediction.

Table S1. Summary of malate dehydrogenase for medical tapeworm and trematode.

**Class**	**Family**	**Genus**	**SPECIES**	**Sequence summary**	**SEQUENCE ID**
Cestoda	Diphyllobothriidea	*Dibothriocephalus*	*D. latus*	3	DILT_0001486601, DILT_0001648601, DILT_0000453201
		Spirometra	*S. mansoni*	8	*Sm*MDH1_SERJ2_LOCUS4602, SmMDH2_SPER_0002854401, *Sm*MDH3_SPER_0003366401, *Sm*MDH4_SERJ2_LOCUS18211, *Sm*MDH5_TRINITY_DN32812_c0_g1_i1, *Sm*MDH6_TRINITY_DN32812_c0_g1_i9, *Sm*MDH7_TRINITY_DN32812_c0_g1_i10, *Sm*MDH8_TRINITY_DN32812_c0_g1_i11
		Schistocephalus	*S. solidus*	5	SSLN_0000919501, SSLN_0001553101, SSLN_0001884001, SSLN_0001588801, SSLN_0001017101
	Taeniidae	*Echinococcus*	*E. canadensis*	3	EcG7_04628, EcG7_08839, EcG7_08021
			E. granulosus	8	EgrG_00578, EgrG_000893100, EgrG_000517200, EgrG_07281, EgrG_001185000, EgrG_000417100, EgrG_07010, EgrG_08628
			E. oligarthrus	1	Eoli_000517200
			E. multilocularis	4	EmuJ_000893100, EmuJ_001185000, EmuJ_000417100, EmuJ_001185100
		Taenia	*T. asiatica*	9	TASK_TASs00004g01136, TASK_0000488101, TASK_0000266701, TASK_TASs00143g08337, TASK_0000036701, TASK_TASs00010g02429, TASK_0000299001, TASK_TASs00010g02428, TASK_0000644501
			T. multiceps	3	Tm1G001281, Tm1G001282, Tm1G001697
			T. saginata	4	TSAs00003g01040, TSAs00063g07180, TSAs00026g04349, TSAs00026g04348

			T. solium	4	TsM_000048200, TsM_000657300, TsM_000267800, TsM_000460300
		Hydatigera	*H. taeniaeformis*	5	TTAC_0000405101, TTAC_0000780101, TTAC_0000298901, TTAC_0000412301, TTAC_0001013001
	Hymenolepididae	*Hymenolepis*	*H. diminuta*	11	HDID_0000660801, HDID_LOCUS6753, HDID_0000060201, HDID_0000060301, HDID_LOCUS8449, HDID_0000561501, HDID_LOCUS6544.1, HDID_LOCUS6544.2, HDID_LOCUS2678, HDID_LOCUS8540, HDID_0001029401
			H. nana	5	HNAJ_0000045201, HNAJ_0000045301, HNAJ_0000388801, HNAJ_0000594201, HNAJ_0001192401
			H. microstoma	3	HmN_000258300, HmN_000063000, HmN_000063100
	Mesocestoididae	*Mesocestoides*	*M. corti*	11	MCU_0001007601, MCU_008646, MCU_003955_RA, MCU_003955_RB, MCU_006567_RA, MCU_006567_RB, MCU_006567_RC, MCU_009062, MCU_0000749901, MCU_014229, MCU_0000835901
Trematoda	Schistosomatidae	*Schistosoma*	*S. japonicum*	13	Sjp_0041910, Sjp_0024400, Sjp_008117.1, Sjp_008117.2, Sjp_008117.3, Sjp_010981.1, Sjp_010981.2, Sjp_000090.1, Sjp_000090.2, Sjp_000090.3, Sjp_000090.4, Sjp_000090.5, Sjp_000090.6
			S. haematobium	15	SHAE1_78140, SHAE2_80780, SHAE_MS3_00007547.1, SHAE_MS3_00007547.2, SHAE2_30640, SHAE_MS3_00005651.1, SHAE_MS3_00005651.2, SHAE_MS3_00005651.3, SHAE1_29410, SHAE2_57410.1, SHAE2_57410.2, SHAE2_57410.3, SHAE2_57410.4, SHAE1_55450, SHAE_MS3_00010820
			S. mansoni	4	Smp_035270, Smp_047370, Smp_070860.1, Smp_070860.2
			S.margrebowiei	5	SMRG1_76940, SMRG1_28460.1, SMRG1_28460.2, SMRG1_28460.3, SMRG1_54720

			S. mattheei	5	SMTH1_31430.1, SMTH1_31430.2, SMTH1_84930.1, SMTH1_84930.2, SMTH1_60330
			S. rodhaini	11	SRDH2_28760, SRDH1_74570, SRDH1_39570, SRDH1_48420.1, SRDH1_48420.2, SRDH1_48420.3, SRDH2_55100, SRDH2_78190.1, SRDH2_78190.2, SRDH2_78190.3, SRDH2_78190.4
			S.bovis	10	SBOV2_75390.1, SBOV2_75390.2, SBOV1_39370, SBOV2_39230, SBOV_DC041_0008291, SBOV1_51080, SBOV2_51440, SBOV_DC041_0012974, SBOV1_74870, SBOV_DC041_0002698
			S. curassoni	8	SCUR1_28450, SCUR1_75730.1, SCUR1_75730.2, SCUD_0000539301, SCUD_0001337701, SCUD_0001603901, SCUD_0002203501, SCUR1_54230
			S. intercalatum	6	SINT1_75170, SINT2_74490, SINT2_52660, SINT1_54060, SINT2_27250, SINT1_28460
			S. guineensis	4	SGUI1_76760, SGUI1_28050.1, SGUI1_28050.2, SGUI1_54400
			S. spindale	3	SSPN1_73470.1, SSPN1_73470.2, SSPN1_27270
			S. turkestanicum	3	STRK1_73010, STRK1_51400, STRK1_27030,
		Trichobilharzia	*T. regenti*	4	TREG1_105430, TREG1_70880, TREG1_48600.1, TREG1_48600.2
			T. szidati	5	TSZD1_103560, TSZD1_71860, TSZD1_103540, TSZD1_103580, TSZD1_103500
		Heterobilharzia	*H. americana*	5	HAMR1_95450, HAMR2_84560, HAMR2_48220, HAMR2_58740, HAMR1_66780
	Opisthorchiidae	*Clonorchis*	*C. sinensis*	6	CSKR_110103, CSKR_103326, CSKR_csin102558, CSKR_csin101489, CSKR_csin112591, CSKR_111940

			C. felineus	5	CRM22_008362, CRM22_002201.1, CRM22_002201.2, CRM22_008422.1, CRM22_008422.2
			C. viverrini	3	T265_16243, T265_04260, T265_05466
	Fasciolidae	*Fasciolopsis*	*F. buski*	3	FBUS_11060, FBUS_08099, FBUS_02962
		Fasciola	*F. gigantica*	7	FGIG_07754, FGIG_06624, FGIG_06623, FGIG_07755, FGIG_00665, FGIG_08365, FGIG_05200
			F. hepatica	6	Fhe_859_0.47, Fhe_377_0.75, Fhe_D915_009078, Fhe_120_0.22, Fhe_D915_010544, Fhe_D915_004345
	Paragonimidae	*Paragonimus*	*P. westermani*	1	DEA37_0012094
	Echinostomatidae	*Echinostoma*	*E. caproni*	3	ECPE_0000453101, ECPE_0001285701, ECPE_0001684501
	Microphallidae	*Atriophallophorus*	*A. winterbourni*	3	jcf_7180000222489_0.19, jcf_7180000242691_0.4, jcf_7180000271253_0.4
	Dicrocoeliidae	*Dicrocoelium*	*D. dendriticum*	6	DDND1_112850.1, DDND1_112850.2, DDND1_112850.3, DDND1_112850.4, DDND1_112850.5, DDND1_32360
	Gyrodactylidae	*Gyrodactylu*s	*G. salaris*	1	scf_7180006950882_0.1

Table S2. The putative motifs of *Sm*MDH.

Motif	Consensus sequence	Length	No. of MDH proteins	e-value
1	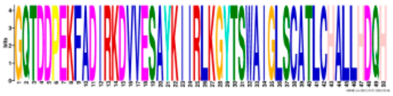 GQTDDPEKFADIRKDVVESAYKIIRLKGYTSWAIGLSCATLCHALLHDQH	50	4	4.8e-105
2	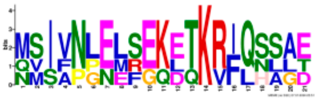 MSIVNLELSEKETKRIQSSAE	21	8	1.6e-043<br>1.6e-043
3	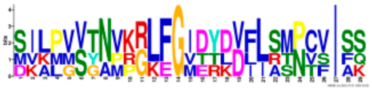 SILPVVTNVKGLFGIDYDVFLSMPCVISS	29	8	1.1e-052
4	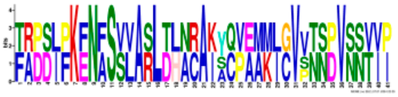 TRPDJPKFNFSVLARLDHARAKYQPEAKJGVVTNPVNNVIP	41	4	2.5e-020
5	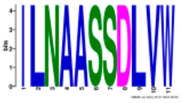 ILNAASSDLVW	11	4	7.6e-011
6	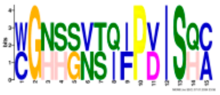 WGNHGNSQFPDISQA	15	4	8.5e-011
7	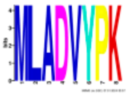 MLADVYPK	8	4	2.7e-008
8	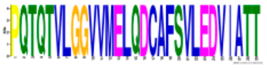 PQTQTVLGGVVMELQDCAFSVLEDVIATT	29	2	1.3e-006
9	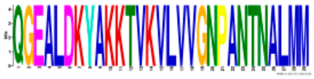 QGEALDKYAKKTVKVLVVGNPANTNALMM	29	2	8.6e-005
10	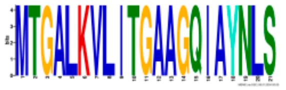 MTGALKVLITGAAGQIAYNLS	21	2	3.1e-001

Table S3. Primers sequences designed for qRT-PCR.

Gene	Primer name	Sequence (5'-3')	Product size (bp)
SERJ2_LOCUS4602	SERJ2_LOCUS4602-F<br>SERJ2_LOCUS4602-R	CCAAGAAGACTGTGAAGGT<br>GTGGGAGATGTCAGGAAAC	233
SPER_0002854401	SPER_0002854401-F<br>SPER_0002854401-R	AATCGCCTACAATCTTTCC<br>CATTACAACACCACCCAAG	121
SPER_0003366401	SPER_0003366401-F<br>SPER_0003366401-R	GTTGCCTCCCTGACTCTT<br>TACGCACATCCAAACCCT	271
	SERJ2_LOCUS18211-F	ATTGCCGCCGAACTGATG	230
SERJ2_LOCUS18211	SERJ2_LOCUS18211-R	AACTGCTCACGCTCCTCC	
TRINITY_DN32812_c0_g1_i1	TRINITY_DN32812_c0_g1_i1F<br>TRINITY_DN32812_c0_g1_i1R	CGATAGCCCAAGAGGTGT<br>GCATTTCAGTTGCCGTAT	174
TRINITY_DN32812_c0_g1_i9	TRINITY_DN32812_c0_g1_i9R	GCCGATAGCCCAAGAGGT<br>GAGCGTTGCCGTATGGAG	172
TRINITY_DN32812_c0_g1_i10	TRINITY_DN32812_c0_g1_i10F<br>TRINITY_DN32812_c0_g1_i10R	GCCGATAGCCCAAGAGGT<br>GAGCGTTGCCGTATGGAG	172
GAPDH	GAPDH-F<br>GAPDH-R	AGCAACCTCGTTGATGTCGT<br>TGAATTGACCGTGGGTGGAG	97

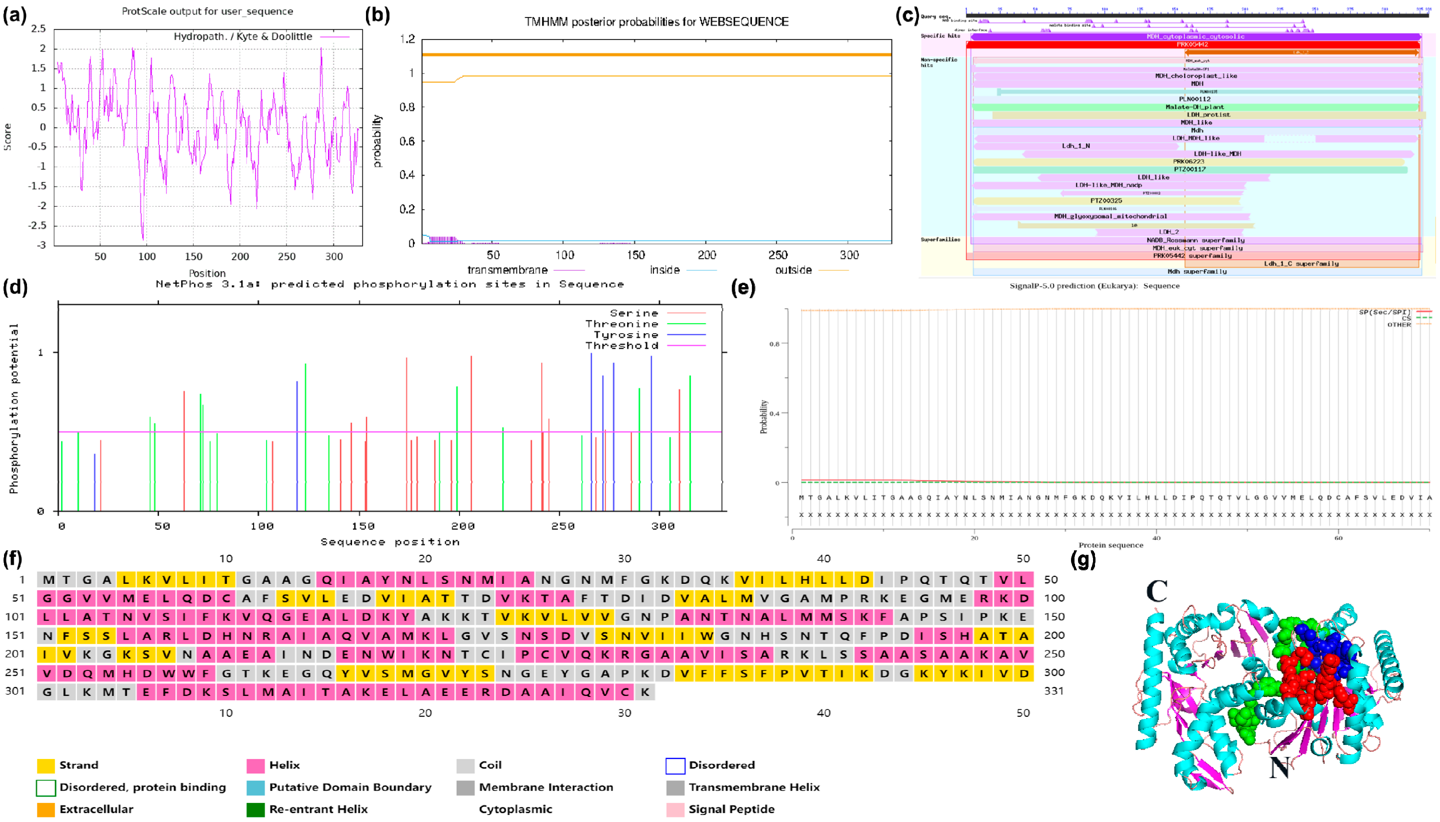

Figure S1. *Sm*MDH1 structural and functional prediction results. (a) hydrophobicity prediction; (b) transmembrane prediction; (c) functional structure domain prediction; (d) phosphorylation site prediction; (e) signal peptide prediction; (f) secondary structure prediction, the yellow indicates strand, the purple represents helix; (g) 3D structure prediction.
